# Association Between Non-alcoholic Fatty Liver Disease and Subclinical Atherosclerosis Assessed by Carotid Intima-Media Thickness: A Systematic Review and Meta-Analysis

**DOI:** 10.7759/cureus.112324

**Published:** 2026-07-09

**Authors:** Nasrin Akter, Mst Fatema Firoz, Farzana Akter Shifa, Kabbya Kumar Datta, Tasnuva Islam, Md Zahidul Alam Khan Sykot, Md Akib Akram, Md Khaledur Rahman, Md Atik Shahryar

**Affiliations:** 1 Department of Medicine, Dhaka Medical College and Hospital, Shahbag, Dhaka, BGD; 2 Department of Emergency Medicine, Nova Maldives, Male, MDV; 3 Department of Medicine, Shaheed Ziaur Rahman Medical College and Hospital, Bogura, BGD; 4 Department of Medicine, Shaheed Suhrawardy Medical College and Hospital, Dhaka, BGD; 5 Department of Medicine, Uttara Adhunik College and Hospital, Uttara, Dhaka, BGD

**Keywords:** atherosclerosis, carotid intima-media thickness, meta-analysis, non-alcoholic fatty liver disease, systematic review, ultrasonography

## Abstract

Non-alcoholic fatty liver disease (NAFLD) is variably associated with subclinical atherosclerosis, measured using carotid intima-media thickness (CIMT), although findings across studies remain inconsistent. This systematic review and meta-analysis aimed to evaluate the association between NAFLD and subclinical atherosclerosis, assessed by CIMT, and to explore possible sources of heterogeneity. A literature search covering almost 25 years (January 2000 to March 2026) was conducted. Included studies reported CIMT measurements in adults diagnosed with NAFLD using either liver biopsy or ultrasonography. A random-effects model was used to calculate pooled effect sizes using Hedges’ g. Subgroup analyses were performed based on geographic region (Asia vs Europe) and diagnostic modality for NAFLD. Eight studies comprising 4,384 patients with NAFLD and 2,843 controls were included. The pooled effect size showed a significant increase in CIMT among patients with NAFLD (Hedges’ g = 0.79; 95% CI: 0.42 to 1.17). Considerable heterogeneity was observed (I² = 94.13%). Studies using ultrasonography demonstrated a smaller effect size (Hedges’ g = 0.66; p = 0.001) compared with those using liver biopsy (Hedges’ g = 1.36). A borderline significant difference was observed between European populations (Hedges’ g = 1.12) and Asian populations (Hedges’ g = 0.64; p = 0.090). Publication bias was detected; however, after adjustment using the trim-and-fill method, the association remained significant (adjusted Hedges’ g = 0.62; 95% CI: 0.36 to 0.88). Overall, NAFLD was significantly associated with increased CIMT, suggesting a higher burden of subclinical atherosclerosis in affected individuals. Future studies should employ standardized diagnostic criteria and uniform measurement protocols to reduce heterogeneity.

## Introduction and background

Non-alcoholic fatty liver disease (NAFLD) is currently the most common chronic liver disease worldwide, affecting approximately 25% of the adult population [1]. The disease spectrum ranges from simple steatosis to non-alcoholic steatohepatitis (NASH), progressing further to fibrosis, cirrhosis, and hepatocellular carcinoma [2]. In addition to hepatic manifestations, NAFLD has been increasingly associated with cardiovascular disease (CVD) [3]. Importantly, cardiovascular complications represent the leading cause of mortality in patients with NAFLD rather than liver-related outcomes [4]. The proposed pathophysiological links between NAFLD and atherosclerosis include systemic inflammation, insulin resistance, oxidative stress, and atherogenic dyslipidemia [3,4].

Subclinical atherosclerosis refers to early structural changes in the arterial wall that are detectable in the absence of clinical symptoms. Carotid intima-media thickness (CIMT) is a validated, non-invasive surrogate marker of subclinical atherosclerosis and is independently associated with myocardial infarction and stroke risk [4]. Increased CIMT reflects both adaptive intimal thickening and the presence of early atherosclerotic changes, including plaque formation [5]. Several studies have reported an association between NAFLD and increased CIMT; however, findings have been inconsistent, likely due to variations in study design, patient populations, diagnostic criteria for NAFLD, and adjustment for confounding variables [4,6].

Cross-sectional and cohort studies have demonstrated significantly higher CIMT values in patients with NAFLD compared with non-NAFLD controls, even after adjustment for traditional cardiovascular risk factors [7,8]. In contrast, other studies have reported that this association becomes attenuated or non-significant after adjustment for metabolic syndrome components or insulin resistance [9,10]. Additional variability may arise from differences in CIMT measurement protocols, including assessment of the common carotid artery (CCA) versus the internal carotid artery, as well as differences in reporting mean versus maximum CIMT values [11]. Furthermore, effect estimates may vary depending on the diagnostic modality used for NAFLD, such as liver biopsy (gold standard), ultrasonography, or biomarker-based definitions [12].

A previous meta-analysis suggested a positive association between NAFLD and CIMT; however, it included a limited number of studies and did not adequately address the substantial heterogeneity observed [7]. Since its publication, several larger and higher-quality studies have been conducted, warranting an updated synthesis of the evidence. In addition, important subgroup analyses such as stratification by NAFLD diagnostic modality, CIMT measurement site, and adjustment for key confounders including body mass index (BMI) and Homeostatic Model Assessment for Insulin Resistance (HOMA-IR) have not been comprehensively explored. 

It is also relevant to note that the terminology of NAFLD is being replaced with metabolic dysfunction-associated steatotic liver disease (MASLD) in recent literature. While the present review used the term NAFLD to align with the included studies, this evolving nomenclature should be considered in future research. Therefore, the primary objective of this systematic review and meta-analysis was to evaluate the association between NAFLD and subclinical atherosclerosis, as assessed by CIMT, and to investigate potential sources of heterogeneity and publication bias.

## Review

Methodology

This systematic review and meta-analysis were conducted in accordance with the Preferred Reporting Items for Systematic Reviews and Meta-Analyses (PRISMA) guidelines [13].

Description of Search Strategy

A comprehensive literature search was performed across five electronic databases: PubMed, Web of Science, Scopus, Cochrane Library, and Embase, covering the period from January 2000 to March 2026. The search strategy incorporated three main concept groups combined using Boolean operators: NAFLD-related terms (including Medical Subject Headings [MeSH]/Emtree terms and text words such as “non-alcoholic fatty liver disease,” “NAFLD,” and “fatty liver”), CIMT-related terms (including “carotid intima-media thickness,” “CIMT,” and “intima-media thickness”), and atherosclerosis-related terms (including “atherosclerosis” and “subclinical atherosclerosis”).

To ensure inclusion of contemporary evidence reflecting current definitions of NAFLD, filters were applied for human studies, English language, adult populations (≥18 years), and publication type limited to original articles. The publication year range was restricted to 2000 to March 2026. Database-specific search syntax was adapted accordingly: PubMed used exploded MeSH terms and [tiab] field restrictions; Web of Science used Topic (TS) searches with wildcards; Scopus used TITLE-ABS-KEY fields without proximity operators; and the Cochrane Library used MeSH terms and title/abstract/keyword searches with automatic word variation. This structured approach was designed to maximize sensitivity while maintaining specificity.

The detailed search string for PubMed was: ("non-alcoholic fatty liver disease"[MeSH] OR "NAFLD"[tiab] OR "fatty liver"[tiab]) AND ("carotid intima-media thickness"[MeSH] OR "CIMT"[tiab] OR "intima media thickness"[tiab] OR "carotid atherosclerosis"[tiab]) AND ("atherosclerosis"[MeSH] OR "subclinical atherosclerosis"[tiab]). All other databases also used the same search string.

In addition to electronic database searching, manual searching was performed to identify studies not captured electronically and to screen grey literature sources. Reference lists of all included full-text articles and relevant systematic reviews were also hand-searched. Manual screening was conducted independently by two reviewers. Any disagreements regarding eligibility were resolved through discussion or consultation with a third reviewer. Although no language or publication restrictions were applied during the search process, only studies with available full-text articles in English were included in the final analysis. Manual searching identified three additional records, of which one met the inclusion criteria and was included in the final dataset.

Description of Eligibility Criteria

Eligibility criteria were defined using the Population, Intervention/Exposure, Comparison, and Outcomes (PICO) framework to ensure transparency and reproducibility [14].

The study population included adults aged 18 years or older, regardless of sex or cardiometabolic risk profile. Individuals with known heavy alcohol consumption or other established causes of hepatic steatosis were excluded to ensure diagnostic specificity for NAFLD. Regarding exposure, NAFLD diagnosis was accepted if confirmed using validated imaging modalities or liver biopsy. Studies defining NAFLD solely based on elevated liver enzymes without evidence of hepatic steatosis were excluded to avoid misclassification bias (Table 1).

**Table 1:** PICO-based eligibility criteria for study inclusion in the meta-analysis CIMT: carotid intima-media thickness; NAFLD: non-alcoholic fatty liver disease; PICO: Population, Intervention/Exposure, Comparison, and Outcomes [14].

PICO Component	Inclusion criteria	Exclusion criteria
Population (participants)	Adults (≥18 years) of any sex or ethnicity, with or without metabolic comorbidities (obesity, diabetes, hypertension, dyslipidemia)	Individuals <18 years; pregnant women; patients with secondary causes of hepatic steatosis (alcohol >30g/day men, >20g/day women; hepatitis B/C; Wilson’s disease; drug-induced steatosis)
Intervention/exposure	Diagnosis of NAFLD by any validated method: liver biopsy (gold standard), abdominal ultrasonography (hepatic steatosis grade ≥1), computed tomography, magnetic resonance imaging (MRI), magnetic resonance spectroscopy (MRS), or fatty liver index. The NAFLD definition required the exclusion of significant alcohol intake and other liver diseases.	NAFLD diagnosed solely by elevated liver enzymes without imaging or biopsy; studies mixing NAFLD with other liver diseases without separate subgroup data
Comparison	Non-NAFLD control group (no hepatic steatosis on imaging or biopsy, or healthy individuals without known liver disease)	Studies without a non-NAFLD comparator; single-arm studies
Outcome	Carotid intima-media thickness measured by B-mode ultrasound at common carotid artery (CCA), bulb, or internal carotid artery (ICA), reported as mean, maximum, or mean of maximum CIMT (mm). Subclinical atherosclerosis is defined by either an absolute CIMT value or a≥75th percentile for age/sex.	CIMT measured exclusively by methods other than ultrasound (e.g., MRI); studies reporting only plaque presence without CIMT values; no quantitative CIMT data for meta-analysis
Study design	Observational studies: cross-sectional, case-control, nested case-control, or prospective/retrospective cohort studies	Randomized controlled trials (unexposed controls not applicable), case reports, case series (<10 participants), reviews, editorials, animal studies, in vitro studies

Data Extraction

A standardized data extraction form was developed in advance and piloted on three randomly selected studies. Two reviewers independently extracted data from all included studies. The following variables were collected: first author’s name, year of publication, country of study, study design, sample size (number of patients with NAFLD and controls), mean age, proportion of male participants, diagnostic method for NAFLD (e.g., biopsy, ultrasonography, magnetic resonance imaging), CIMT measurement site (common carotid artery, carotid bulb, or internal carotid artery), type of CIMT measurement (mean, maximum, or mean of maximum values with standard deviations), adjustment for confounding variables, and baseline metabolic characteristics including BMI, fasting glucose, insulin resistance (HOMA-IR), lipid profile, and blood pressure.

For studies reporting multiple NAFLD severity categories (e.g., simple steatosis and non-alcoholic steatohepatitis), data were extracted separately where applicable. Any discrepancies between reviewers were resolved through consensus or consultation with a third reviewer. Corresponding authors were contacted up to two times over three weeks to obtain missing data, including standard deviations or correlation coefficients.

Quality Assessment and Publication Bias

Methodological quality of included observational studies was assessed using the Risk of Bias in Non-randomized Studies of Exposure (ROBINS-E) tool [15]. Two independent reviewers evaluated each study across seven domains, categorizing risk of bias as low, moderate, serious, or critical. Studies rated as having serious or critical risk of bias were included in sensitivity analyses to assess their influence on pooled estimates.

Publication Bias Assessment

Publication bias was evaluated using funnel plot visualization of standardized mean differences (SMD) against standard error (SE). Small-study effects were assessed using Egger’s linear regression test, with a p-value < 0.10 considered indicative of statistically significant asymmetry [16]. When publication bias was detected, adjusted pooled estimates were calculated using the trim-and-fill method proposed by Duval and Tweedie [17].

Statistical Analysis

The SMD with 95% confidence intervals (CI) was used as the primary effect size measure, as CIMT was reported using different arterial segments (common, bulb, or internal carotid artery) and measurement approaches (mean, maximum, or mean of maximum CIMT).

For each study, Cohen’s d was calculated using the formula:

\[ d = \frac{M_1 - M_2}{SD_{\text{pooled}}} \]

where M₁ and M₂ represent mean CIMT values for NAFLD and control groups, respectively. The pooled standard deviation (SD_pooled) was calculated as:

\[ SD_{\text{pooled}}= \sqrt{ \frac{ (n_1-1)SD_1^2+(n_2-1)SD_2^2 }{ n_1+n_2-2 } } \]

Cohen’s d was adjusted for small sample bias using Hedges’ correction factor:

\[ J = 1 - \frac{3}{4(n_1 + n_2 - 2) - 1} \] and the effect size (Hedges’ g) was used for meta-analysis. The variance of Hedges’ g was computed as:

\[ V_g = \frac{n_1 + n_2}{n_1 n_2} + \frac{g^2}{2(n_1 + n_2)} \]

with SE calculated as the square root of variance.

For studies reporting multiple effect sizes (e.g., right and left CIMT, mean and maximum IMT, or separate NAFLD severity subgroups), a fixed-effects within-study pooling was performed to generate a single summary effect size per study before inclusion in the primary meta-analysis. This approach avoided double-counting of participants and violations of the independence assumption.

Given expected heterogeneity among studies, pooled estimates were calculated using a random-effects model (DerSimonian-Laird method) with estimation of between-study variance (τ²) [18]. Heterogeneity was assessed using the I² statistic and interpreted as low (25%), moderate (50%), and high (75%).

Subgroup analyses were predefined based on geographic region (Asia vs Europe) and NAFLD diagnostic method (liver biopsy vs ultrasonography) [19]. All statistical tests were two-tailed, and p < 0.05 was considered statistically significant.

Results

Study Selection Process

The database search identified 73 records. No additional records were obtained from registries, organizational sources, websites, or citation searching. After removal of 38 duplicates, 35 records remained. Of these, 15 records were excluded based on title and abstract screening for irrelevant content, leaving 20 records for full-text assessment.

During full-text screening, two articles could not be retrieved. The remaining 18 full-text articles were assessed for eligibility, of which 10 were excluded: one due to an ineligible population (absence of appropriate NAFLD-control comparison) and nine due to non-quantitative reporting of CIMT as a continuous variable.

**Figure 1:**
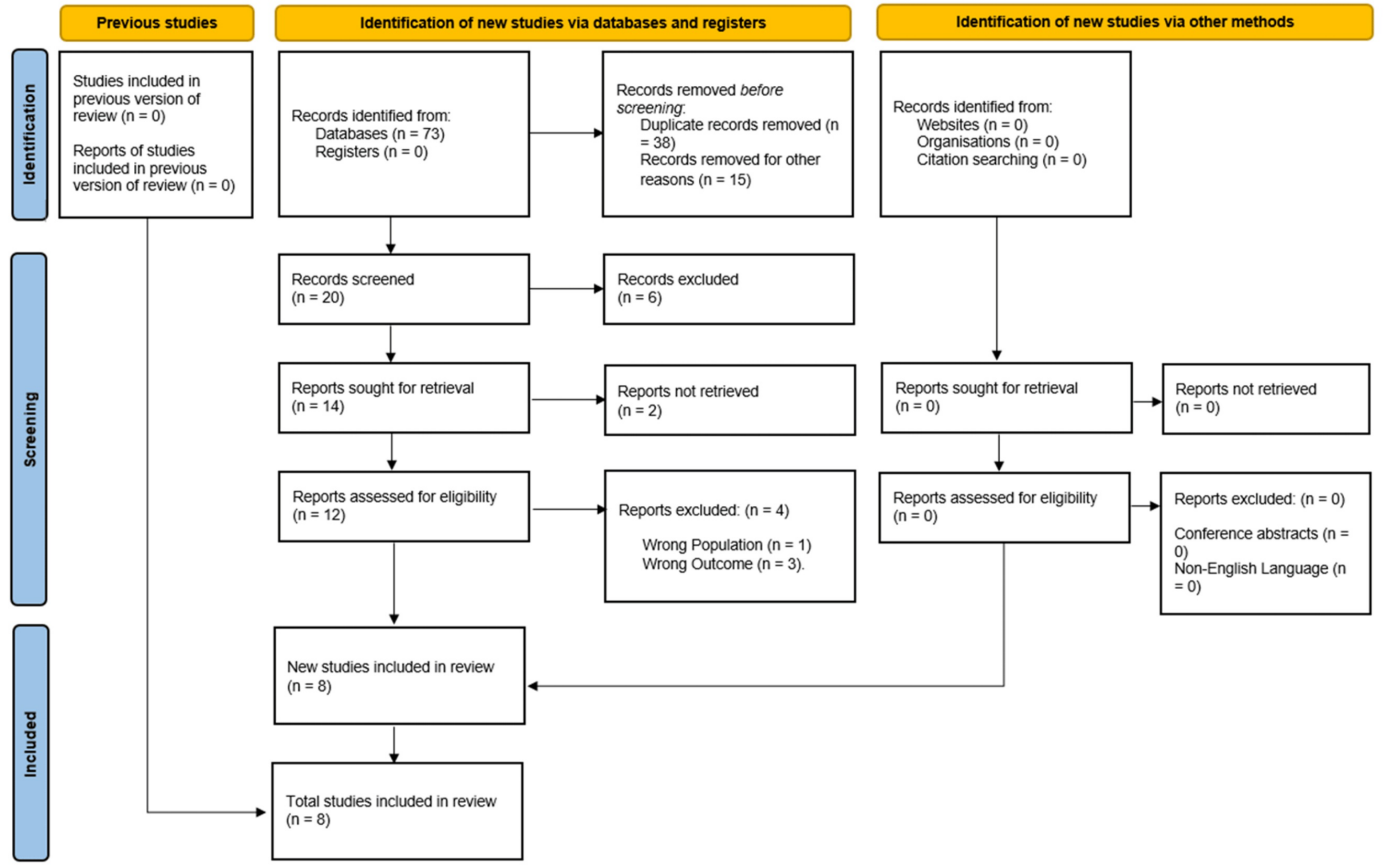
PRISMA flow diagram: study selection process for the systematic review PRISMA: Preferred Reporting Items for Systematic Reviews and Meta-Analyses [13]

Ultimately, eight studies met the inclusion criteria and were included in the final systematic review and meta-analysis (Figure 1) [20-27].

Study Characteristics

Eight observational studies comprising 4,384 patients with NAFLD and 2,843 controls were included in the meta-analysis. The studies were conducted across seven countries: Turkey, China, South Korea (two studies), India, Algeria, Italy, and Iran. Regarding study design, two studies were case-control studies [20,25], three were cross-sectional studies with a case-control design [24,26,27], and three were cross-sectional studies [21-23].

Two studies used liver biopsy, the gold standard for NAFLD diagnosis [20,25], whereas the remaining six studies used abdominal ultrasonography, with one study additionally incorporating FibroScan (Echosens, Paris, France) assessment. All studies measured CIMT at the CCA. However, measurement protocols varied slightly, with some studies reporting mean CIMT, others reporting maximum CIMT, and some assessing the right and left carotid arteries separately. Mean CIMT among patients with NAFLD ranged from 0.62 mm [26] to 1.26 mm [25], whereas mean CIMT among controls ranged from 0.50 mm [26] to 0.84 mm [25] (Table 2).

**Table 2:** Characteristics of included studies for meta-analysis: NAFLD diagnosis, CIMT measurement sites, and mean CIMT values in cases and controls CCA: common carotid artery; CIMT: carotid intima-media thickness; L: left carotid artery; mm: millimeter; n: number of participants; NAFLD: non-alcoholic fatty liver disease; NASH: non-alcoholic steatohepatitis; R: right carotid artery.

Study	Country	Design	NAFLD diagnosis	CIMT site	Cases (n)	Controls (n)	Mean CIMT Cases (mm)	Mean CIMT Controls (mm)
Aygun et al., 2008 [20]	Turkey	Case-control	Liver biopsy	CCA	40	40	0.646	0.544
Zhang et al., 2016 [21]	China	Cross-sectional	Ultrasonography	CCA	123	599	0.81	0.69
Kim et al., 2014 [22]	South Korea	Cross-sectional	Ultrasonography	CCA	3226	1211	0.844	0.786
Karnati et al., 2026 [23]	India	Cross-sectional	Ultrasonography	CCA	75	76	Right: 1.01, Left: 1.02	Right: 0.63, Left: 0.62
Taharboucht et al., 2021 [24]	Algeria	Case-control	Ultrasonography + FibroScan	CCA (R & L)	213	213	Right: 0.65, Left: 0.65	Right: 0.57, Left: 0.58
Targher et al., 2005 [25]	Italy	Case-control	Liver biopsy	CCA far wall	50	40	Simple: 0.96, NASH: 1.26	0.84
Mohammadzadeh et al., 2019 [26]	Iran	Case-control	Ultrasonography	CCA (posterior wall, both sides)	150	150	0.62	0.50
Kim et al., 2009 [27]	South Korea	Cross-sectional	Ultrasonography	CCA (mean of max from both sides)	507	514	Males: 0.77, Females: 0.78	Males: 0.71, Females: 0.70

The CIMT measurement protocol was generally consistent across studies, with the exception of one study that measured CIMT on the lateral wall of the common carotid artery. All studies adjusted for age and sex, and most additionally adjusted for BMI, smoking status, blood pressure, and lipid parameters. Two studies adjusted for HOMA-IR [20,25], while the study by Kim et al. [22] further adjusted for diabetes duration and history of vascular disease. All eight included studies adjusted for age and sex in their multivariate analyses, confirming that the observed associations were independent of these basic demographic factors.

Across nearly all studies, patients with NAFLD demonstrated less favorable metabolic profiles than controls, including higher BMI, fasting glucose, HOMA-IR, triglyceride levels, and systolic blood pressure, as well as lower high-density lipoprotein (HDL) cholesterol levels. However, some notable exceptions were reported. Mohammadzadeh et al. [26] found comparable prevalences of hyperlipoproteinemia and hypertension between groups, while Taharboucht et al. [24] reported similar mean fasting glucose levels despite a statistically significant between-group difference (Table 3).

**Table 3:** Detailed characteristics of included studies: CIMT measurement protocols, adjustment for confounders, and key baseline metabolic parameters *Kim et al., 2014 [22]: For the primary analysis, the extreme-group contrast (NAFLD with insulin resistance versus no NAFLD without insulin resistance) was used to minimize confounding by insulin resistance. This subgroup included 1,742 NAFLD cases and 698 controls. ** Karnati et al., 2026 [23] and Taharboucht et al., 2021 [24]: Right and left CIMT values were averaged before pooling to avoid double-counting participants. *** Targher et al., 2005 [25]: Simple steatosis and NASH subgroups were pooled using a fixed-effects within-study model to generate a single effect size per study. **** Kim et al., 2009 [27]: Male and female subgroups were pooled using a fixed-effects within-study model to generate a single effect size per study. AIP: atherogenic index of plasma, ALT: alanine aminotransferase, AST: aspartate aminotransferase, ATP III: Adult Treatment Panel III, BMI: body mass index, BP: blood pressure, CCA: common carotid artery, CIMT: carotid intima-media thickness, DBP: diastolic blood pressure, FLI: fatty liver index, GGT: gamma-glutamyl transferase, HDL: high-density lipoprotein, HLP: hyperlipoproteinemia, HOMA-IR: Homeostatic Model Assessment for Insulin Resistance, hsCRP: high-sensitivity C-reactive protein, HTN: hypertension, IMT: intima-media thickness, IQR: interquartile range, IR: insulin resistance, LDL: low-density lipoprotein, MetS: metabolic syndrome, NAFLD: non-alcoholic fatty liver disease, NASH: non-alcoholic steatohepatitis, PWV: pulse wave velocity, SBP: systolic blood pressure, SD: standard deviation, TC: total cholesterol, TG: triglycerides, M: men, W: women, mmol/L: millimoles per liter, mg/dL: milligrams per deciliter, g/L: grams per liter, mmHg: millimeters of mercury, kg/m²: kilograms per square meter, μU/mmol: microunits per millimole, NR: Not Reported.

Study	CIMT Site	CIMT Type	CIMT Cases (mm, mean ± SD)	CIMT Controls (mm, mean ± SD)	Adjustment for Confounders	BMI Cases (kg/m²)	BMI Controls (kg/m²)	Fasting Glucose Cases	Fasting Glucose Controls	HOMA-IR Cases	HOMA-IR Controls	Triglycerides Cases	Triglycerides Controls	HDL Cases (mg/dL)	HDL Controls (mg/dL)	SBP Cases (mmHg)	SBP Controls (mmHg)
Aygun et al., 2008 [20]	CCA	Mean IMT	0.646 ± 0.091	0.544 ± 0.067	Age, sex, BMI, smoking, BP, lipids, HOMA-IR, NAFLD diagnosis	31.2 ± 4.7	25.6 ± 3.8	98.6 ± 28.3 mg/dL	91.3 ± 12.1 mg/dL	5.8 ± 4.6	1.94 ± 0.88	192.6 ± 157.2 mg/dL	117.2 ± 52.8 mg/dL	45.4 ± 7.1	49.1 ± 15.4	143 ± 20	124 ± 17
Zhang et al., 2016 [21]	CCA (10-20 mm proximal to bifurcation)	Mean CIMT	0.81 ± 0.25	0.69 ± 0.18	Age, sex, BMI, waist, SBP, DBP, TC, TG, LDL, HDL, MetS, ALT, AST, GGT, hsCRP, antihypertensive/lipid-lowering drugs	23.5 ± 3.6	21.4 ± 3.7	8.57 ± 3.85 mmol/L	8.48 ± 4.00 mmol/L	NR	NR	1.71 ± 1.80 mmol/L	0.93 ± 0.59 mmol/L	1.24 ± 0.39 mmol/L	1.44 ± 0.40 mmol/L	125.5 ± 13.9	122.1 ± 15.8
Kim et al., 2014 [22]*	CCA (1 cm proximal to bulb)	Mean CIMT	0.844 ± 0.004 (NAFLD+IR)	0.786 ± 0.008 (no NAFLD+insulin sensitive)	Age, sex, waist, diabetes duration, smoking, vascular disease history, hypoglycemic/antihypertensive/lipid-lowering agents, SBP, HDL, log triglycerides	25.4 ± 3.2	22.7 ± 2.4	9.08 mmol/L	7.56 mmol/L	1.51 (Kitt %/min)	3.29 (Kitt %/min)	2.00 mmol/L	1.21 mmol/L	1.25 mmol/L	1.39 mmol/L	138.3	130.2
Karnati et al., 2026 [23]**	CCA (1 cm proximal to bifurcation)	Mean IMT (averaged R & L)	1.015 ± 0.38	0.625 ± 0.085	Age, sex, BMI, triglycerides, HDL-C, AIP, FLI (multivariate regression for NAFLD severity)	28.06 ± 3.94	25.9 ± 4.42	NR	NR	NR	NR	205.68 ± 70.52 mg/dL	105.49 ± 33.19 mg/dL	30.57 ± 8.06	39.62 ± 6.59	NR	NR
Taharboucht et al., 2021 [24]**	CCA (averaged R & L)	Mean IMT (averaged R & L)	0.65 ± 0.1	0.575 ± 0.1	Age, sex, smoking, abdominal obesity, overall obesity, SBP, nocturnal DBP, dyslipidemia, elevated GGT/ALT, HOMA≥3, CRP≥6 mg/L, IMT>75th, PWV>90th, carotid plaques	31.2 ± 4.4	27.8 ± 4.9	0.9 ± 0.1 g/L	0.9 ± 0.1 g/L	2.4 ± 1.6 μU/mmol	1.2 ± 1.6 μU/mmol	1.4 ± 1.1 g/L	1.0 ± 0.4 g/L	0.4 ± 0.1 g/L	0.4 ± 0.1 g/L	132.6 ± 18.7	127.9 ± 17.4
Targher et al., 2005 [25]***	CCA far wall	Mean IMT (pooled simple steatosis + NASH)	1.12 ± 0.22	0.84 ± 0.13	Age, sex, HOMA-IR, MetS (ATP III)	26.6 ± 1.6	26.2 ± 1.8	NR	NR	4.14 ± 2.05	1.70 ± 1.0	NR	NR	NR	NR	NR	NR
Mohammadzadeh et al., 2019 [26]	CCA (posterior wall, both sides)	Mean IMT	0.62 ± 0.19	0.50 ± 0.13	Age, BMI, smoking, hyperlipoproteinemia, diabetes, hypertension	29.1 ± 4.5	28.1 ± 5.0	NR	NR	NR	NR	HLP%: 47.4%	HLP%: 50.9%	NR	NR	HTN%: 49.3%	HTN%: 50.2%
Kim et al., 2009 [27]****	CCA (~1 cm proximal to bulb, mean of max IMT)	Mean IMT (pooled males + females)	0.775 ± 0.23	0.705 ± 0.20	Age, sex, waist circumference, SBP, fasting glucose, total/HDL-C ratio, smoking, alcohol	25.75 ± 2.9	23.45 ± 2.6	5.36 mmol/L (median)	5.05 mmol/L (median)	2.92 (median)	1.90 (median)	2.12 mmol/L (median)	1.41 mmol/L (median)	NR	NR	133.2 ± 17.7	127.5 ± 18.6

Comparative Risk of Bias Assessment

**Figure 2:**
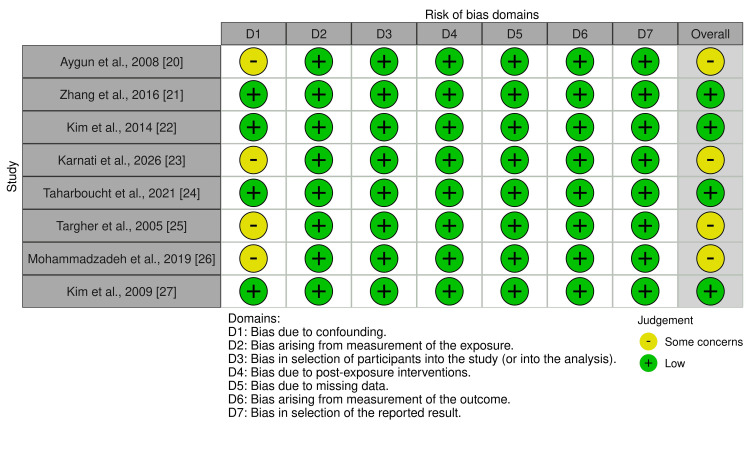
Interpretation of ROBINS-E risk of bias assessment for included studies. ROBINS-E: Risk of Bias in Non-randomized Studies of Exposure [15]

Overall, all eight studies were judged to have a low risk of bias across six of the seven ROBINS-E domains. Four studies were rated as having "some concerns" in Domain 1 (bias due to confounding) [20,23,25,26]. This rating reflected incomplete adjustment for important metabolic confounders, including HOMA-IR, fasting glucose, and blood pressure, or insufficient reporting of these variables in the extracted data (Figure 2).

Publication Bias

**Figure 3:**
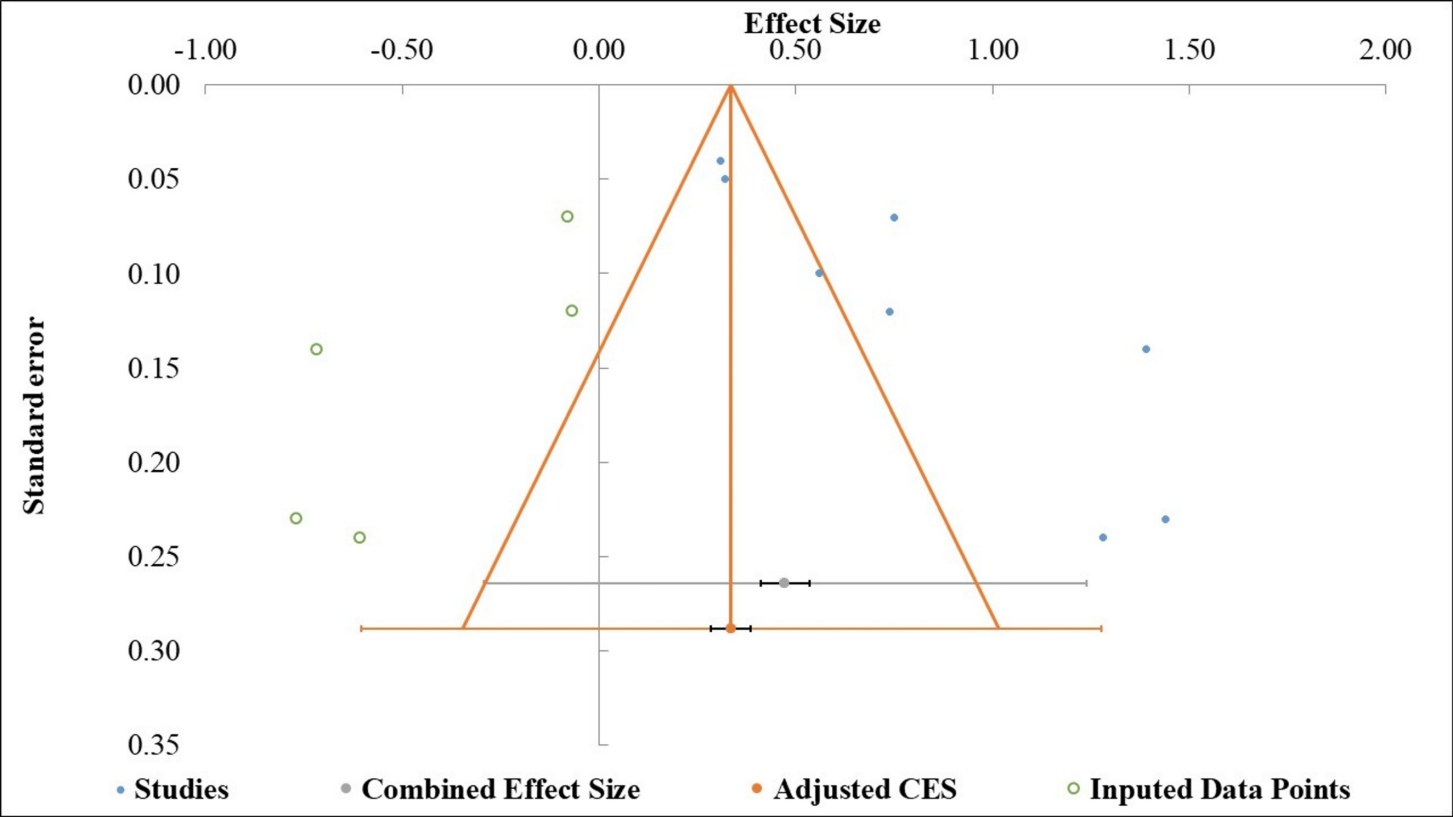
Funnel plot for publication bias assessment of studies examining the association between NAFLD and CIMT CIMT: carotid intima-media thickness; NAFLD: non-alcoholic fatty liver disease; CES: combined effect size.

Egger's regression test revealed funnel plot asymmetry (intercept: 6.55, 95% CI: 3.11 to 9.99, p = 0.004), suggesting possible publication bias. However, it is important to note that Egger's test may have limited power with only eight included studies, and the finding of asymmetry should be interpreted cautiously. Visual inspection of the funnel plot should be prioritized over statistical testing in small meta-analyses (Figure 3, Table 4). As prespecified in the study protocol, a trim-and-fill analysis was performed to evaluate the potential impact of publication bias by imputing hypothetical missing studies and recalculating the pooled effect estimate.

**Table 4:** Egger's regression test of effect size against standard error for publication bias assessment Egger's regression test [16]

Parameter	Estimate	Std. Error	95% CI-Lower limit	95% CI-Upper limit
Intercept	6.55	1.45	3.11	9.99
Slope	0.06	0.11	-0.19	0.31
t-value	4.51			
p-value	0.004			

However, because only eight studies were included, the statistical power of Egger’s test was limited. Therefore, these findings should be interpreted cautiously. A sensitivity analysis excluding the three smallest studies was also performed to evaluate the robustness of the pooled effect estimate.

Duval and Tweedie’s Trim-and-Fill Analysis

The trim-and-fill procedure [17] imputed three potentially missing studies on the left side of the funnel plot, representing smaller studies with null or negative associations between CIMT and NAFLD. Following adjustment, the pooled Hedges’ g decreased from 0.79 (95% CI: 0.42-1.17) to 0.62 (95% CI: 0.36-0.88).

Despite this reduction, the adjusted effect size remained statistically significant (p = 0.003) and represented a moderate positive association. These findings suggest that publication bias may have moderately inflated the original estimate but did not fully account for the observed association between NAFLD and subclinical atherosclerosis. Consequently, the adjusted estimate (Hedges’ g = 0.62, 95% CI: 0.36-0.88) was considered a more conservative approximation of the true effect and is presented alongside the primary analysis.

Sensitivity Analysis

After excluding the three smallest studies (total sample size <150 participants) [20,23,25], the pooled Hedges’ g was 0.54 (95% CI: 0.37-0.71), based on five studies comprising 4,219 patients with NAFLD and 2,727 controls. Exclusion of only the smallest study [20] yielded a pooled Hedges’ g of 0.76 (95% CI: 0.48-1.04).

All sensitivity analyses remained statistically significant and demonstrated moderate positive associations, supporting the robustness of the primary findings. The progressive reduction in effect size from 0.79 to 0.54 following sequential exclusion of smaller studies suggests the presence of small-study effects and possible publication bias. Nevertheless, even the most conservative estimate indicated a clinically meaningful difference in CIMT between patients with NAFLD and controls.

Meta-analysis findings

Forest Plot and Heterogeneity Assessment

The forest plot demonstrated a significant association between NAFLD and subclinical atherosclerosis, as measured by CIMT, with a pooled Hedges’ g of 0.79 (95% CI: 0.42-1.17). Substantial heterogeneity was observed across studies (I² = 94.13%). In addition, the prediction interval ranged from -0.06 to 1.65, crossing the null value and indicating considerable variability in effect sizes across study populations and settings.

**Figure 4:**
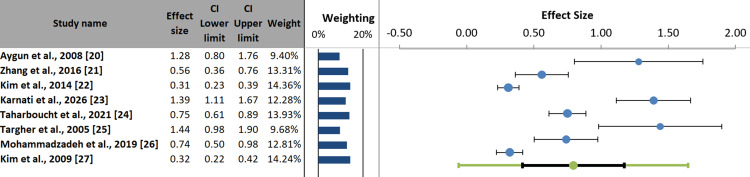
Forest plot of the association between NAFLD and CIMT. CIMT: carotid intima-media thickness; NAFLD: non-alcoholic fatty liver disease.

These findings suggest that the pooled estimate should be interpreted with caution and support further exploration of heterogeneity through subgroup analyses (Figure 4).

Subgroup Analysis by NAFLD Diagnostic Method

**Figure 5:**
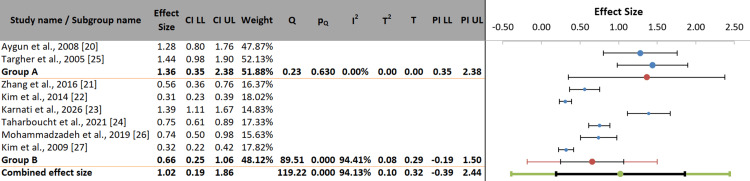
Subgroup analysis of the association between NAFLD and CIMT stratified by NAFLD diagnostic method (liver biopsy vs ultrasonography). CIMT: carotid intima-media thickness; LL: lower limit; NAFLD: non-alcoholic fatty liver disease, UL: upper limit.

Subgroup analysis based on NAFLD diagnostic modality (liver biopsy versus ultrasonography) is presented in Figure 5. The overall pooled effect size across subgroups was 1.02 (95% CI: 0.19-1.86), with a prediction interval ranging from -0.39 to 2.44.

A statistically significant difference was observed between diagnostic subgroups (between-group Q* = 11.72; degrees of freedom = 1; p = 0.001). Studies using liver biopsy demonstrated a larger effect size (Hedges’ g = 1.36) than studies using ultrasonography (Hedges’ g = 0.66). Residual heterogeneity remained substantial within the ultrasonography subgroup (I² = 94.41%), indicating that additional factors, such as CIMT measurement protocols, study design, geographic location, and confounder adjustment strategies, likely contributed to between-study variability.

Diagnostic modality explained 57.77% of the between-study variance, indicating that approximately 42% of the heterogeneity remained unexplained and warranted further investigation.

Subgroup Analysis by Geographic Region

**Figure 6:**
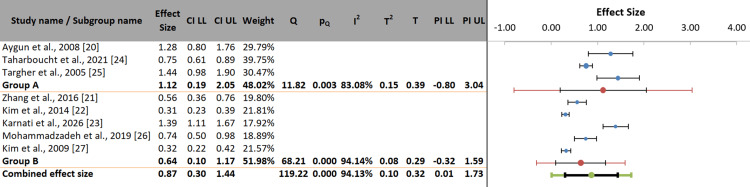
Subgroup analysis of the association between NAFLD and CIMT stratified by geographic region (Europe vs. Asia) CIMT: carotid intima-media thickness; LL: lower limit; NAFLD: non-alcoholic fatty liver disease, UL: upper limit.

A subgroup analysis stratified by geographic region was performed to evaluate differences in the association between NAFLD and CIMT (Figure 6). The pooled effect size across regional subgroups was 0.87 (95% CI: 0.30-1.44), with a prediction interval of 0.01-1.73 based on 7,227 participants.

There was a trend toward significant differences between geographic regions (between-group Q* = 2.87; degrees of freedom = 1; p = 0.090), although this did not reach the conventional threshold for statistical significance. Residual heterogeneity was not statistically significant after accounting for geographic region (within-group Q = 9.77; degrees of freedom = 6; p = 0.135). 

Although the between-group difference was not statistically significant, effect sizes differed meaningfully across regions. The pooled effect size was larger in European/Mediterranean populations (Hedges’ g = 1.12) than in Asian populations (Hedges’ g = 0.64), suggesting potential regional variation in the relationship between NAFLD and subclinical atherosclerosis (Figure 6).

Discussion

In this systematic review and meta-analysis, we found a significant positive association between NAFLD and subclinical atherosclerosis, as measured by CIMT. The pooled Hedges’ g of 0.79 (95% confidence interval (CI): 0.42-1.17) indicates that individuals with NAFLD had moderately higher CIMT values than those without NAFLD. These findings are consistent with those of Sookoian and Pirola [7], who also reported a positive association between NAFLD and CIMT. However, given the cross-sectional and case-control designs of the included studies, causal inference cannot be established. The findings should be interpreted as an association rather than causation.

Several biological mechanisms may explain the observed association between NAFLD and increased CIMT. As described by Targher et al. [3], insulin resistance, a hallmark feature of NAFLD, contributes to systemic inflammation, oxidative stress, and atherogenic dyslipidemia. Together, these processes promote endothelial dysfunction and arterial wall thickening, which are reflected by increased CIMT measurements. In addition, Lonardo et al. [5] suggested that NAFLD may directly contribute to cardiovascular disease through hepatic production of pro-inflammatory cytokines, including interleukin-6 and tumor necrosis factor-α.

Considerable variability was observed across the included studies. Targher et al. [25] reported one of the largest effect sizes (Hedges’ g = 1.44), possibly because NAFLD was diagnosed using liver biopsy and the study included patients with non-alcoholic steatohepatitis (NASH). Similarly, Aygun et al. and Karnati et al. reported large effect sizes (1.28 and 1.39, respectively), which may be attributable to smaller sample sizes and stricter inclusion criteria. In contrast, Kim et al. [22,27] reported smaller, although still statistically significant, effect sizes (0.31 and 0.32, respectively). These differences may reflect larger sample sizes, more comprehensive adjustment for confounding factors, and inclusion of relatively healthier study populations.

The substantial heterogeneity observed in this analysis (I² = 94.13%) is consistent with previous systematic reviews. Sookoian and Pirola [7] similarly reported marked interstudy heterogeneity and emphasized the importance of subgroup analyses to identify potential sources of variation. In the present study, subgroup analyses based on NAFLD diagnostic modality and geographic region explained 57.77% and 22.73% of the between-study variance, respectively.

Studies using liver biopsy demonstrated a significantly larger effect size (Hedges’ g = 1.36) than those using ultrasonography (Hedges’ g = 0.66; p = 0.001). This finding supports the observations of Musso et al. [12], who suggested that ultrasonography may fail to detect mild steatosis, potentially leading to misclassification of some individuals with NAFLD as controls and attenuation of observed associations. Liver biopsy remains the reference standard for diagnosing and staging NAFLD and provides more accurate classification of disease severity. Moreover, both biopsy-based studies [20,25] included patients with NASH, a subgroup known to have a higher cardiovascular risk profile than individuals with simple steatosis.

Although not statistically significant, regional differences were observed between European/Mediterranean populations (Hedges’ g = 1.12) and Asian populations (Hedges’ g = 0.64; p = 0.090). Genetic factors may partly explain this trend. For example, the PNPLA3 I148M variant, which has been associated with more severe NAFLD and increased cardiovascular risk, is more prevalent in some European populations. Differences in baseline metabolic characteristics may also contribute. In the study by Targher et al. [25], control participants had a higher mean BMI than controls in the Korean study by Kim et al. [22], suggesting that the relative contribution of NAFLD to increased CIMT may vary across populations with different underlying metabolic risk profiles.

Publication bias was detected using Egger’s test (p = 0.004). However, after adjustment using the Duval and Tweedie trim-and-fill method [17], the association remained statistically significant (Hedges’ g = 0.62, 95% CI: 0.36-0.88). These findings suggest that publication bias may have modestly inflated the original effect estimate but does not fully explain the observed association between NAFLD and subclinical atherosclerosis. Similar concerns regarding publication bias have been reported in previous meta-analyses of NAFLD and CIMT [7], highlighting the importance of prospective study registration and publication of negative findings.

Sensitivity analyses further supported the robustness of the results. After exclusion of the three smallest studies, the pooled effect size remained significant (Hedges’ g = 0.54, 95% CI: 0.37-0.71). Although this reduction suggests the presence of small-study effects, even the most conservative estimate indicated a clinically meaningful increase in CIMT among patients with NAFLD. Previous evidence suggests that each 0.1 mm increase in CIMT is associated with an approximately 15% increase in the risk of myocardial infarction and stroke [4].

The findings of this study are also consistent with research using other surrogate markers of atherosclerosis. The Coronary Artery Risk development in Young Adults (CARDIA) study demonstrated associations between NAFLD and increased coronary artery calcium scores [28], as well as impaired flow-mediated dilation [29]. The consistency of findings across different vascular markers strengthens the evidence supporting an independent association between NAFLD and subclinical atherosclerosis.

Major strengths of this meta-analysis include the inclusion of biopsy-confirmed NAFLD studies, the comprehensive search of five databases, the use of the ROBINS-E tool for quality assessment, and the conduct of predefined subgroup analyses. Nevertheless, several limitations should be considered when interpreting these findings.

Study limitations

Several limitations should be acknowledged. First, substantial statistical heterogeneity was observed among the included studies (I² = 94.13%), and considerable residual heterogeneity remained within the ultrasonography subgroup (I² = 94.41%). Second, most studies (six of eight) diagnosed NAFLD using ultrasonography rather than liver biopsy, which may have introduced misclassification bias and underestimated the true association. Third, evidence of publication bias was identified (Egger’s test, p = 0.004), suggesting that smaller studies with null or negative findings may be underrepresented in the literature. Fourth, the included studies were predominantly cross-sectional or case-control in design, limiting the ability to establish temporal or causal relationships between NAFLD and subclinical atherosclerosis. Fifth, variations in CIMT measurement protocols, including differences in arterial segments assessed, reporting of mean versus maximum CIMT, and operator-dependent measurement techniques, may have contributed to measurement bias and heterogeneity. Sixth, the relatively small number of included studies limited the scope of subgroup analyses and reduced the precision of some subgroup estimates. Seventh, the absence of prospective protocol registration on PROSPERO may limit transparency and reproducibility, although the review was conducted in accordance with PRISMA 2020 guidelines and all methods were predefined. Eighth, Egger's test for publication bias may be underpowered with fewer than 10 studies, and therefore, the finding of significant asymmetry should be interpreted with caution. Finally, most studies were conducted in Asian and European populations, which may limit the generalizability of the findings to other geographic and ethnic populations. All included studies were observational in design (cross-sectional or case-control), which precluded the establishment of a causal relationship between NAFLD and subclinical atherosclerosis. Reverse causality cannot be excluded.

Future directions

Future research should focus on large prospective cohort studies with long-term follow-up to clarify the temporal relationship between NAFLD progression and the development of subclinical atherosclerosis and cardiovascular events. Greater use of reference-standard diagnostic methods, including liver biopsy and advanced imaging modalities such as magnetic resonance spectroscopy and magnetic resonance imaging proton density fat fraction, is warranted.

Standardized CIMT measurement protocols should also be adopted to improve comparability across studies. Individual participant data meta-analyses may help address residual confounding through adjustment for factors such as age, sex, BMI, HOMA-IR, blood pressure, lipid parameters, smoking status, and medication use.

Future studies should investigate whether NAFLD severity, assessed using fibrosis markers such as the Fibrosis-4 Index (FIB-4) [30], NAFLD Fibrosis Score (NFS) [31], elastography, or histological staging, modifies the association with CIMT. Additional research is also needed in underrepresented regions, including North America, South America, and Africa, to improve the global applicability of current findings.

Mechanistic studies evaluating inflammatory biomarkers, oxidative stress markers, and gut-derived metabolites may further clarify the biological pathways linking NAFLD and atherosclerosis. In addition, randomized controlled trials should evaluate whether interventions that improve NAFLD, including lifestyle modification, pioglitazone, vitamin E, and glucagon-like peptide-1 receptor agonists, can reduce CIMT progression or promote regression of subclinical atherosclerosis. Finally, future studies should assess whether incorporating CIMT into established cardiovascular risk prediction models improves risk stratification among individuals with NAFLD.

## Conclusions

This systematic review and meta-analysis demonstrated a significant association between NAFLD and increased CIMT, a validated marker of subclinical atherosclerosis. Individuals with NAFLD consistently exhibited higher CIMT values than those without NAFLD, suggesting a greater burden of early atherosclerotic changes. Subgroup analyses indicated that the strength of this association varied according to the method used to diagnose NAFLD, with studies employing more accurate diagnostic approaches generally reporting stronger associations. Although regional differences were observed, these findings were not conclusive. The association remained consistent across publication bias adjustments and sensitivity analyses, supporting the overall robustness of the findings. Taken together, the available evidence suggests that NAFLD is associated with subclinical atherosclerosis and may contribute to an increased cardiovascular risk profile. However, the substantial heterogeneity among studies and the observational nature of the included evidence warrant cautious interpretation. Further well-designed prospective studies using standardized assessment protocols and accurate diagnostic methods are needed to clarify the relationship between NAFLD and subclinical atherosclerosis and to determine its implications for cardiovascular risk assessment and clinical practice.
